# Study on the Aging Effects of Relative Humidity on the Primary Chemical Components of Palm Leaf Manuscripts

**DOI:** 10.3390/polym17010083

**Published:** 2024-12-31

**Authors:** Wenjie Zhang, Shan Wang, Hong Guo

**Affiliations:** 1 Key Laboratory of Archaeomaterials and Conservation, Ministry of Education, Institute of Cultural Heritage and History of Science & Technology, University of Science and Technology Beijing, Beijing 100083, China; d202310769@xs.ustb.edu.cn; 2 Chinese Academy of Cultural Heritage, Beijing 100029, China; cnicpbj@gmail.com

**Keywords:** palm leaf manuscripts, cellulose, infrared spectra, simulated aging experiment, preventive conservation

## Abstract

Palm Leaf Manuscripts represent a significant component of the world’s cultural heritage. Investigating their primary chemical components and understanding the transformations these materials undergo under environmental influences are crucial for elucidating their material characteristics and aging mechanisms and developing effective strategies for preventive conservation. This study utilized infrared absorption spectroscopy and X-ray diffraction analysis to examine changes in the primary chemical components of Palm Leaf Manuscripts under varying relative humidity conditions over extended periods. The findings reveal that dry environments lead to surface cracking, while humid environments promote mold growth, both of which contribute to the degradation of the primary chemical components. These degradative processes reduce cellulose crystallinity and thermal stability. The deterioration is particularly severe under high humidity, with hemicellulose degrading faster and more extensively than cellulose under the same conditions. After 200 days of aging at 10% RH and 90% RH, cellulose degradation reached 19.82% and 54.40%, respectively, while hemicellulose degradation was 34.78% and 64.28%. Correspondingly, the relative crystallinity of cellulose decreased by 8.01% and 13.11%. In contrast, samples maintained at 50% RH exhibited minimal deterioration, with cellulose and hemicellulose degrading by only 4.08% and 13.55%, respectively, and a 6.61% reduction in cellulose crystallinity. These results suggest that a relative humidity of 50% is optimal for the preservation of Palm Leaf Manuscripts. This study offers significant insights into the ageing mechanisms and preventive conservation of Palm Leaf Manuscripts, as well as other cellulose-based organic heritage materials.

## 1. Introduction

Before the widespread adoption of paper, Palm Leaf Manuscripts were a prominent medium for literature across South and Southeast Asia. These manuscripts not only preserved knowledge across a wide range of disciplines—including history, literature, philosophy, art, and science—but also played a pivotal role in the dissemination of Buddhism, embodying profound cultural and historical significance [1,2,3]. Consequently, they are recognized as invaluable cultural heritage, necessitating efforts for their restoration and preventive conservation.

Significant advancements have been made in the study of Palm Leaf Manuscripts. Researchers have conducted comprehensive analyses of their material composition [4,5,6,7] and production techniques [8,9], carried out preliminary investigations into the degradation processes affecting these materials [10], and categorized the common types of deterioration observed [11,12]. Based on these findings, various restoration and conservation strategies have been developed [13,14]. Furthermore, substantial progress has been achieved in cataloguing and deciphering the texts contained within these manuscripts [15,16,17]. These efforts have provided critical foundational data for the scientific understanding and preventive conservation of Palm Leaf Manuscripts, making a significant contribution to their long-term preservation and restoration.

Despite these advances, research on the impact of environmental factors and the mechanisms underlying the aging process of Palm Leaf Manuscripts remains limited. Several studies have investigated the effects of environmental factors on the color, mechanical properties, and hygroscopicity of Palm Leaf Manuscripts [4,18,19,20]. However, the majority of these studies have primarily focused on the aging characteristics of already deteriorated artifacts or the influence of environmental conditions on specific properties, such as mechanical performance and hygroscopicity. Although some research has examined changes in the chemical composition of Palm Leaf Manuscripts, there remains a lack of in-depth investigation into the processes and mechanisms driving alterations in their chemical structure and primary chemical components during aging.

Similar to paper and wood, Palm Leaf Manuscripts are cellulose-based organic artifacts primarily composed of cellulose, hemicellulose, and lignin. Alterations in their chemical structure and primary chemical components can significantly affect key properties such as color, mechanical performance, hygroscopicity, and stability, all of which play a crucial role in their preservation and restoration. These chemical structural changes are closely linked to the storage environment. Environmental factors—including temperature [21,22,23], humidity, light exposure [24,25,26], and air pollutants [27,28]—can exert substantial effects. For example, high temperatures and light exposure can induce thermal oxidation and photooxidation of cellulose and hemicellulose, resulting in discoloration of the material. Furthermore, air pollutants interacting with moisture can accelerate the acidic hydrolysis of cellulose, thereby exacerbating its degradation.

Controlled indoor environments, such as libraries and artifact storage rooms, are typically effective in mitigating the effects of environmental factors like temperature, light exposure, and air pollutants. However, relative humidity, which is more challenging to regulate and prone to fluctuations, emerges as the most significant environmental factor impacting Palm Leaf Manuscripts and other cellulose-based materials, such as paper and wood. Variations in humidity can lead to physical deformation, causing irreversible structural damage [29], while high humidity levels promote microbial growth and proliferation [30]. These conditions not only alter the chemical structure and primary components of the manuscripts but also accelerate their deterioration and impair other key properties. In addition, the optimal humidity conditions for preserving Palm Leaf Manuscripts are often determined by referencing preservation methods for similar materials, such as paper, rather than being based on research specifically tailored to Palm Leaf Manuscripts. As a result, there is a pressing need to investigate the primary chemical components of these manuscripts and how they change under different relative humidity conditions to better understand their intrinsic properties, aging mechanisms, and preservation strategies.

This study focuses on initially processed Palm Leaf Manuscripts to examine the effects of varying relative humidity levels on their chemical structure and primary chemical components during aging. The primary innovation of this research lies in elucidating the processes and mechanisms underlying the chemical structural and compositional changes in Palm Leaf Manuscripts under different humidity conditions. Based on the findings, this study proposes optimal humidity conditions for the preservation of these manuscripts, offering valuable data to advance the understanding of the deterioration mechanisms and inform strategies for their preventive conservation.

## 2. Materials and Methods

### 2.1. Experimental Materials

The experimental samples used in this study were raw Palm Leaf Manuscripts from Yunnan Province, China. These samples were produced using the traditional palm-leaf manuscript-making process, which is recognized as part of China’s national intangible cultural heritage. The preparation process involved boiling, washing, air drying, trimming, and flattening the leaves of the talipot palm (*Corypha umbraculifera* L.) tree [8], resulting in the materials used for this research.

**Figure 1:**
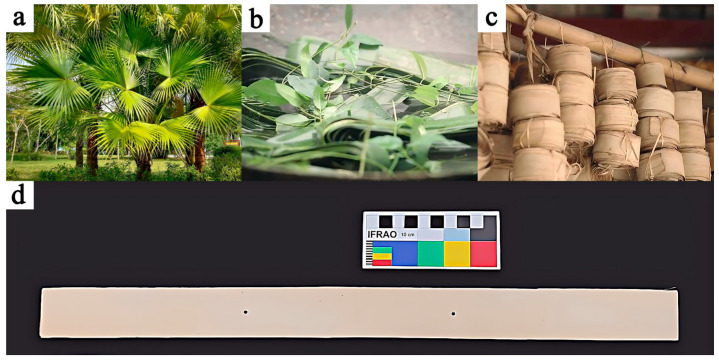
Photograph of the talipot palm tree (**a**); the boiling (**b**) and drying (**c**) step in the traditional processing of palm leaf manuscripts; and the samples used in this study (**d**).

It is important to note that traditional processing techniques may have already altered the chemical structure and primary components of Palm Leaf Manuscripts. For instance, boiling (Figure 1b) is typically conducted to remove chlorophyll from the palm leaves, making them suitable for writing. However, this process may also cause the loss of components such as cellulose. Additionally, acidic substances, such as tamarind and lemons, are often added during boiling to further remove organic impurities like starch and pectin, which helps protect the palm leaves from insect and mold infestation. Nevertheless, residual acidic substances can accelerate the acid hydrolysis of cellulose and other primary components. Similarly, the dehydration of palm leaves during drying (Figure 1c) may damage or even compromise the microscopic structure of cellulose, potentially affecting the long-term preservation of the manuscripts.

Previous studies have shown that the cellulose and hemicellulose of traditionally processed palm leaves degrade significantly compared to fresh palm leaves [18], resulting in a marked reduction in cellulose crystallinity. These findings suggest that traditional processing techniques have already altered the chemical structure and primary components of Palm Leaf Manuscripts, which may, in turn, impact their stability under different preservation environments.

This research focuses on the impact of relative humidity on the chemical structure and primary chemical components of the palm leaves themselves, the core material of palm leaf manuscripts (Figure 1d). Therefore, the raw palm leaf manuscript samples were not subjected to subsequent treatments, such as writing or coloring, in order to avoid any influence from pigments or binding materials on the study results.

### 2.2. Simulated Aging Experiment

**Figure 2:**
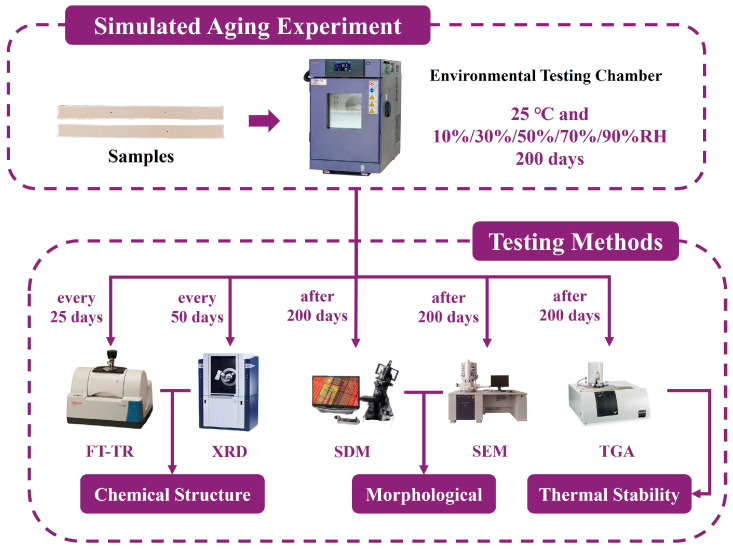
Schematic diagram of the experimental procedure (all instrument images are sourced from the official websites of the manufacturers).

The samples were divided into five groups and placed in an environmental testing chamber (GSH-64, Espec, Osaka, Japan), each subjected to different aging conditions: a constant temperature of 25 °C and relative humidity levels of 10% RH, 30% RH, 50% RH, 70% RH, and 90% RH. Portions of the samples were periodically removed for analysis, with infrared spectroscopy (FT-IR) testing conducted every 25 days of aging and X-ray diffraction (XRD) testing every 50 days. After 200 days of aging, the samples were retrieved for morphological analysis using a super-depth microscope (VHX-6000, KEYENCE, Osaka, Japan). Additionally, some samples were analyzed using scanning electron microscopy (SEM) and thermogravimetric analysis (TGA). The schematic diagram of the simulated aging experiments and other testing procedures is shown in Figure 2.

### 2.3. FT-IR Testing

The aged samples were finely ground and passed through a 200-mesh sieve. Subsequently, they were mixed with KBr (spectrally pure, Macklin, Shanghai, China) at a mass ratio of 1:100 and pressed into pellets. The chemical structure of the samples was analyzed using Fourier transform infrared spectroscopy (FTIR) (Nicolet™ iS™5, Thermo Scientific, Waltham, MA, USA) to compare the differences in characteristic functional groups before and after aging.

To further characterize the changes in the chemical structure of the samples, the infrared spectra were analyzed semi-quantitatively using the peak intensity method. Specific absorption peaks were selected to represent key components: 1730 cm^−1^ (stretching vibration of the carbonyl group in hemicellulose) and 1460 cm^−1^ (stretching vibration of the methylene group in hemicellulose) were used as characteristic peaks for hemicellulose; 1505 cm^−1^ (backbone vibration of butyl propane in butyl lignin) represented lignin; and 1370 cm^−1^ (bending vibration of the methyl group in cellulose) and 1050 cm^−1^ (bending vibration of the glycosidic bond in cellulose) were used as characteristic peaks for cellulose [31,32,33]. The intensity values of these spectral peaks were measured after baseline correction using OMNIC 9.2 software (Thermo Scientific, Waltham, MA, USA).

### 2.4. XRD Testing

The crystal structure and crystallinity of the aged samples were analyzed using an X-ray diffractometer (D8 ADVANCE, Bruker, Munich, Germany). The instrument was operated at a working voltage of 40 kV and a current of 40 mA, with Cu-Kα radiation (wavelength λ = 1.5406 Å) as the radiation source. The scanning angle range was set from 5° to 50° (2θ), with a scanning speed of 1°/min and a step size of 0.02°. The resulting diffraction patterns were fitted and analyzed using MDI Jade 9 software (ICDD, Newtown Square, PA, USA). The crystallinity index (CI) of the samples was calculated using the Segal method.

(1) $$CI=\frac{I_{200}-I_{am}}{I_{200}}\times 100\%$$

where *CI* is the crystallinity index of the sample; $I_{200}$ is the maximum intensity of the lattice diffraction angle of (200) near 2θ = 22.4°, which signifies both the crystalline and non-crystalline regions; and $I_{am}$ is the minimum intensity near the 2θ angle of 18°, indicating the non-crystalline region.

### 2.5. SEM Testing

The samples were gold-coated using a sputter coater (Emitech K550X, Quorum Technologies, Laughton, East Sussex, UK) to enhance their conductivity. The coating process utilized high-purity (99.99%) gold (Au) as the target material. The distance between the samples and the target was maintained at 8 cm, with a sputtering current of 20 mA and a sputtering duration of 60 s. Following the gold-coating process, a field emission scanning electron microscope (Regulus8100, HITACHI, Tokyo, Japan) was employed to examine the microstructure of the samples. The observation parameters were set as follows: an accelerating voltage of 15 kV, a working distance of 15–20 mm, and a magnification range of 200× to 1000×.

### 2.6. TGA Testing

The samples underwent pyrolysis analysis using a thermogravimetric analyzer (Discovery TGA 550, TA Instruments, New Castle, DE, USA) under a nitrogen atmosphere. The gas flow rate was maintained at 20 mL/min, with the temperature ramping from 30 °C to 1000 °C at a heating rate of 20 °C/min. Thermogravimetric (TG) and derivative thermogravimetric (DTG) curves were generated to capture the relationship between temperature and sample mass loss. Parameters such as the weight ratios and pyrolysis temperatures at different stages of the decomposition process were calculated using Origin 2024 software (OriginLab, Northampton, MA, USA).

## 3. Results and Discussion

### 3.1. Aging Results of the Samples

**Figure 3:**
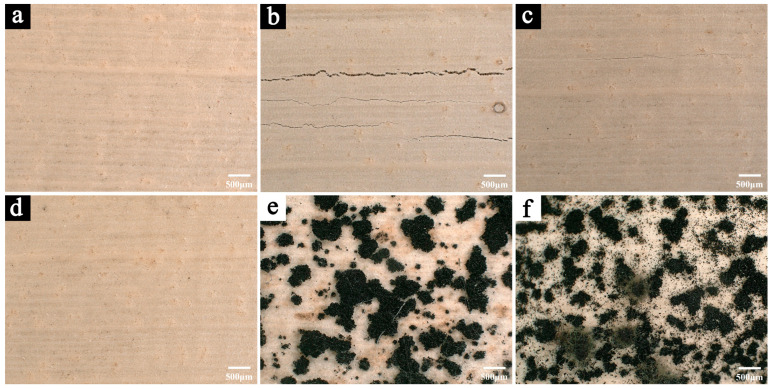
The microscopic images of the samples before aging (**a**) and after 200 days of aging in different humidity conditions: 10% RH (**b**), 30% RH (**c**), 50% RH (**d**), 70% RH (**e**), and 90% RH (**f**).

Figure 3 illustrates the morphological changes of the samples after 200 days of aging under different conditions (temperature maintained at 25 °C, with relative humidity levels of 10% RH, 30% RH, 50% RH, 70% RH, and 90% RH).

After 200 days of aging, surface cracks were observed in the samples exposed to 10% RH (Figure 3b) and 30% RH (Figure 3c), with the cracking being more severe at 10% RH. In contrast, significant mold growth was evident on the surfaces of samples aged at 70% RH (Figure 3e) and 90% RH (Figure 3f), with the latter exhibiting a greater quantity and density of mold. Notably, the samples aged at 50% RH (Figure 3d) showed neither cracking nor microbial deterioration, and their morphology remained largely unchanged compared to the pre-aging state.

It is widely recognized that overly dry environments can cause cellulose-based materials to lose moisture and shrink [29]. Due to the pronounced anisotropy of such materials, differential volume changes during shrinkage in various directions can create internal stresses, resulting in cracking. Conversely, high-humidity environments (typically above 60% RH) provide favorable conditions for mold growth [30], which accelerates the deterioration of cellulose-based organic materials, such as Palm Leaf Manuscripts. These observations suggest that a relative humidity of 50% RH is optimal for preserving Palm Leaf Manuscripts. At this humidity level, excessive drying, which can lead to shrinkage and deformation, is prevented, while excessive moisture that could encourage mold growth is also avoided.

### 3.2. SEM Images of the Samples

**Figure 4:**
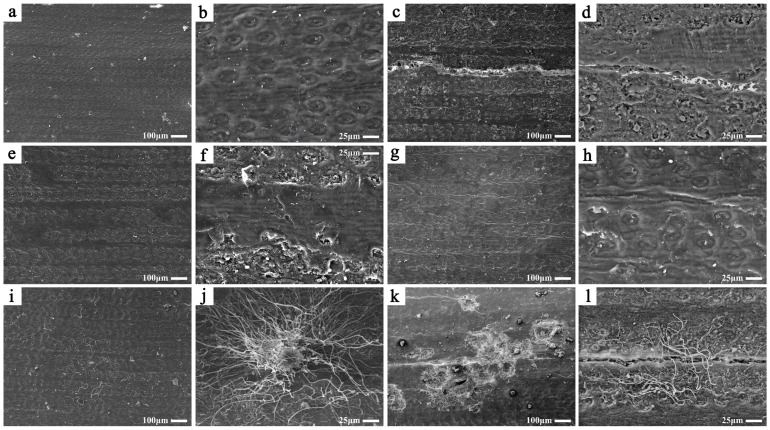
SEM images of the samples before aging (**a**,**b**) and after 200 days of aging under different humidity conditions: 10% RH (**c**,**d**), 30% RH (**e**,**f**), 50% RH (**g**,**h**), 70% RH (**i**,**j**), and 90% RH (**k**,**l**).

Figure 4 presents the microstructural changes observed in the samples after 200 days of aging under different conditions, as captured by scanning electron microscopy (SEM).

Under the scanning electron microscope (SEM), the microstructures of the sample surface, including veins and pores, were clearly visible (Figure 4b). After 200 days of aging at 10% RH (Figure 4c,d) and 30% RH (Figure 4e,f), distinct cracks were observed on the sample surfaces, with the severity of cracking being more pronounced at 10% RH. At this humidity level, transverse cracks spanned nearly the entire surface and reached widths of approximately 20 μm. For samples aged at 70% RH (Figure 4i,j) and 90% RH (Figure 4k,l), images were captured after the majority of surface mold had been removed to prevent contamination of the instrument. Nevertheless, residual mold was still visible on the sample surfaces. SEM images revealed the morphological characteristics of the mold: spherical spores aggregated into clusters, forming networks of hyphae radiating from these spore clusters. Some hyphae had already penetrated the sample surface (Figure 4j). Samples aged at 90% RH exhibited the most severe mold infestation, with numerous mold-induced structures, such as holes and cracks, observed on the surface (Figure 4k). Hyphae extended through these structures, penetrating deeper into the sample’s interior and causing further degradation (Figure 4l). In contrast, samples aged at 50% RH for 200 days showed no signs of cracking or microbial infestation, and their morphology remained largely unchanged compared to their pre-aging state (Figure 4g,h).

In excessively dry environments, cellulose-based materials are prone to deformation due to moisture loss and shrinkage, particularly in materials sensitive to humidity changes. Studies indicate that Palm Leaf Manuscripts share a similar fiber distribution structure with other cellulose-based materials, such as paper and wood. The longitudinal fibers in Palm Leaf Manuscripts are more numerous and densely packed than the transverse fibers, leading to pronounced anisotropy [34]. This anisotropy results in differences in dimensional stability and mechanical performance. Research shows that the mechanical properties of longitudinal fibers are superior to those of transverse fibers [4,35], which explains the formation of transverse cracks during deformation caused by humidity fluctuations [35]. This phenomenon was also observed during the aging process (Figure 4c,d), suggesting that surface cracks are primarily induced by deformation resulting from moisture loss and shrinkage.

Although traditional manufacturing processes remove organic impurities, such as starch and pectin [8]—which are common substrates for mold growth—the primary components of Palm Leaf Manuscripts, including cellulose and hemicellulose, still provide nutrients for mold. When the relative humidity exceeds levels conducive to mold growth (typically above 60% RH), extensive mold colonies form on manuscript surfaces.

These observations suggest that chemical structural changes and the degradation of primary chemical components occur during the aging process under different relative humidity conditions. For instance, surface cracking observed in dry environments indicates partial degradation of primary chemical components during the drying process. This cracking can be attributed not only to the anisotropy of Palm Leaf Manuscripts but also to a decline in their mechanical properties, often linked to the degradation of cellulose and hemicellulose—key components of cellulose-based materials [18]. Additionally, surface cracks may accelerate oxidative degradation reactions in the material’s chemical components. Similarly, samples aged in humid environments exhibited severe mold damage, characterized by extensive pores, cracks, and internal degradation caused by mold hyphae. As aging progresses and mold proliferates, primary chemical components such as cellulose and hemicellulose are consumed as nutrients, further contributing to the material’s deterioration.

### 3.3. Infrared Spectrum of the Samples During the Aging Process

**Figure 5:**
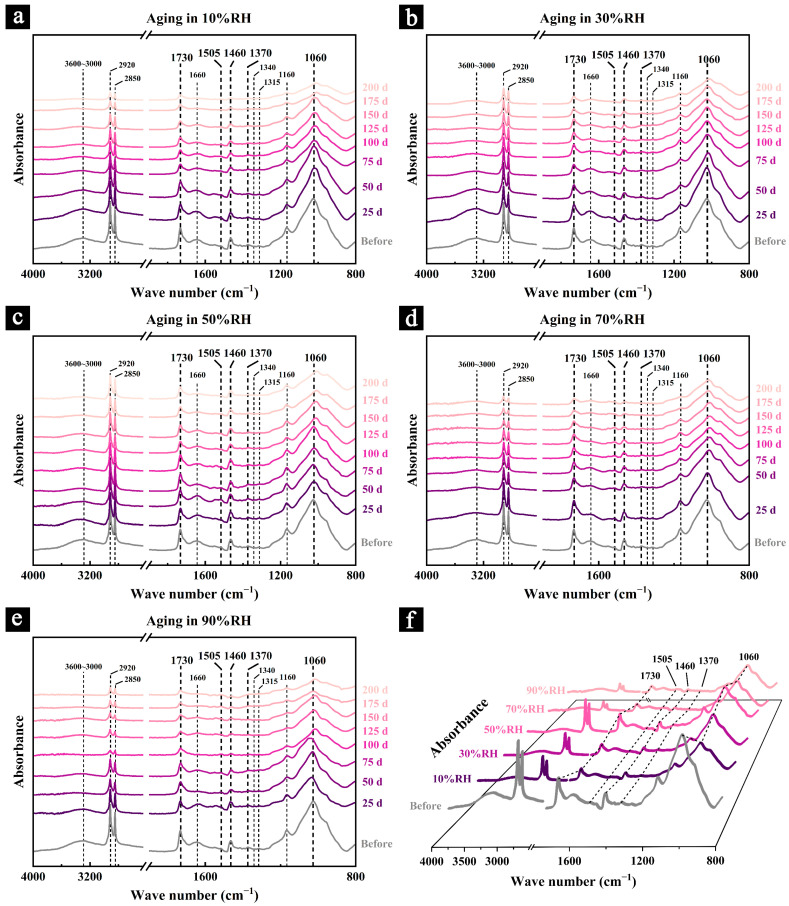
Changes in the infrared spectra of the samples during aging under different humidity conditions: 10% RH (**a**), 30% RH (**b**), 50% RH (**c**), 70% RH (**d**), and 90% RH (**e**); and the infrared spectra of the samples before and after 200 days of aging (**f**).

The infrared spectra of the samples during aging under different conditions and after 200 days of aging are shown in Figure 5. The functional groups and their assignments corresponding to the characteristic peaks and bands in the infrared spectra of the samples are detailed in Table 1.

The infrared spectra of the samples during aging under different conditions revealed varying degrees of reduction in overall absorption intensity and the intensities of characteristic peaks, indicating that the primary chemical components—cellulose, hemicellulose, and lignin—underwent degradation to differing extents under various aging conditions. For instance, as aging progressed, the broad absorption peak at 3600–3000 cm^−1^ diminished in intensity to varying degrees across all samples, with the peak nearly disappearing in those aged at 90% RH. This absorption peak is typically associated with the number of hydroxyl groups and the strength of intra- and intermolecular hydrogen bonding in the primary chemical components, such as cellulose [36]. The decline in intensity suggests significant degradation of hydroxyl groups during aging, accompanied by the collapse of the hydrogen bond network. This degradation likely weakened intermolecular cohesion and substantially reduced the mechanical performance of the material.

The absorption peaks at 2920 cm^−1^ and 2850 cm^−1^, attributed to the stretching vibrations of methyl and methylene groups in cellulose and hemicellulose [37,38], also showed weakening over the course of aging. This reflects the degradation of cellulose and hemicellulose in the samples. Similarly, the reduced intensities of hemicellulose-related peaks at 1730 cm^−1^ and 1460 cm^−1^, along with cellulose-related peaks at 1370 cm^−1^ and 1060 cm^−1^, further indicate the progressive degradation of these components during aging [31,32,33].

Additionally, the characteristic peak at 1060 cm^−1^, primarily associated with the bending vibration of glycosidic bonds in cellulose, exhibited a noticeable shift during aging, with the most pronounced shift observed in samples aged at 90% RH. This peak shift is commonly linked to the degradation of cellulose and hemicellulose, changes in cellulose crystallinity, and alterations in hydrogen bonding within the material. For example, degradation and increased amorphization of cellulose, combined with stronger intermolecular hydrogen bonding, can cause the peak to shift to lower wavenumbers. Conversely, a reduction in hydrogen bonding and increased cellulose crystallinity may result in a shift to higher wavenumbers [39,40]. In the early stages of aging under 90% RH, the broad absorption peak at 3600–3000 cm^−1^ and the hemicellulose characteristic absorption peak at 1730 cm^−1^ exhibited a significant reduction in intensity, while the characteristic peak at 1060 cm^−1^ shifted noticeably to higher wavenumbers. These changes suggest that during the initial stages of mold infestation, the hydroxyl groups in the sample molecules were rapidly lost, hydrogen bonding was weakened, and hemicellulose was preferentially degraded by mold, while cellulose remained relatively stable. As aging progressed, mold infestation began to cause substantial degradation of the cellulose, resulting in the shift of the 1060 cm^−1^ characteristic peak to lower wavenumbers, where it eventually stabilized.

**Table 1:** Characteristic peaks in the infrared spectrum of the samples and corresponding functional group ascriptions.

Wave Number (cm^−1^)	Functional Groups Ascriptions
3600~3000	stretching vibration by the hydroxyl on cellulose and the intermolecular hydrogen bonds [36]
3000~2800	stretching vibration by the methyl and methylene groups on cellulose, hemicellulose, and lignin [37,38]
**1730**	**stretching vibration by the carbonyl group on hemicellulose** [31,32]
1660	stretching vibration by the carbonyl group on deconjugated carbonyl ketone in lignin [37,41]
**1550~1500**	**backbone vibration of butyl propane in butyl lignin** [32,42]
**1460**	**stretching vibration by the methylene group on hemicellulose** [31,32]
1450	bending vibration by the methylene group in lignin and hydroxyl group in cellulose [42,43]
**1370**	**bending vibration by the methyl group on cellulose** [32,33]
1330	bending vibration by the methyl group on methoxy in amorphous cellulose [42,43]
1315	bending vibration by the methylene group on crystalline cellulose [42,44]
1160	bending vibration by the glycosidic bond on glucopyranose, carbohydrate, and crystalline cellulose [38,42]
**1060**	**bending vibration by the glycosidic bond on cellulose** [32,33]

The primary chemical components of the samples exhibited varying degrees of degradation under different aging conditions. Samples aged in humid environments (70% RH and 90% RH) experienced severe structural and morphological damage due to mold invasion (Figure 4k). The primary chemical components, such as cellulose and hemicellulose, underwent significant degradation. Conversely, samples aged in excessively dry environments (10% RH and 30% RH) avoided microbial deterioration, but the rapid loss of moisture created internal stress concentrations, making the cellulose more susceptible to mechanical breakage [18]. This phenomenon was evident macroscopically as surface cracking, as confirmed by the SEM images (Figure 4d,f). The large fractures and microcracks on the surface further exposed cellulose and hemicellulose to external factors such as temperature and oxygen, thereby accelerating their degradation [45,46].

Samples aged at 50% RH maintained the best overall condition, showing no visible deterioration (Figure 3d and Figure 4h). Apart from minor hydroxyl loss and natural aging, the infrared spectra of these samples retained relatively strong absorption intensities throughout the aging process (Figure 5c), indicating minimal degradation of the primary chemical components.

**Figure 6:**
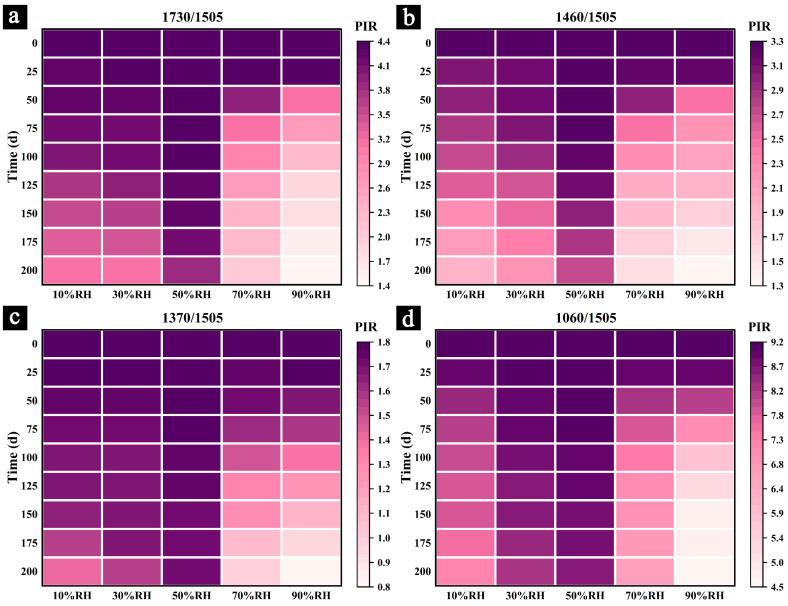
Relative intensity changes in characteristic peaks for cellulose and hemicellulose during the aging process under different humidity conditions: I_1730_/I_1505_ (**a**), I_1460_/I_1505_ (**b**), I_1370_/I_1505_ (**c**), and I_1060_/I_1505_ (**d**).

**Figure 7:**
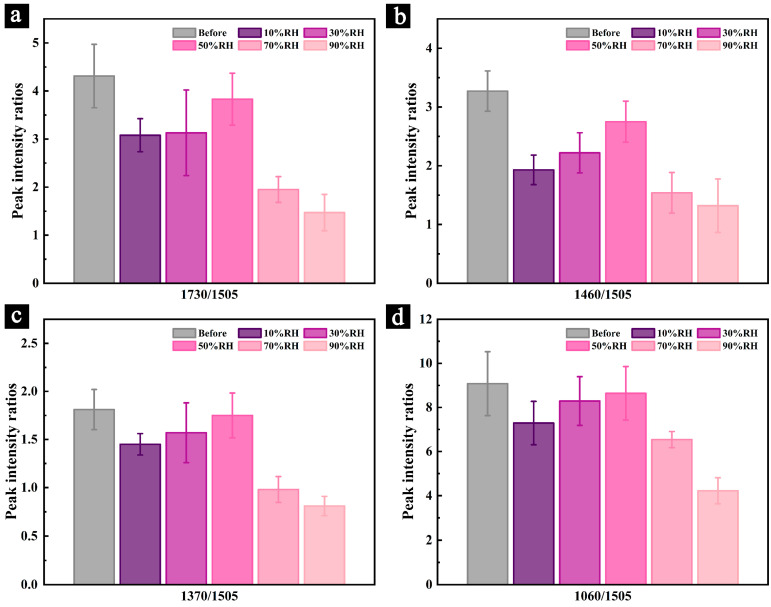
Relative intensities of cellulose and hemicellulose characteristic peaks before and after 200 days of aging: I_1730_/I_1505_ (**a**), I_1460_/I_1505_ (**b**), I_1370_/I_1505_ (**c**), and I_1060_/I_1505_ (**d**).

To further investigate changes in the chemical structure and primary components, a semi-quantitative analysis of the infrared spectra was conducted. Figure 6 and Figure 7 illustrate the changes in the relative intensities of the characteristic peaks for cellulose and hemicellulose during the aging process and after 200 days of aging under different conditions.

The results indicate that the relative intensities of the characteristic peaks for cellulose and hemicellulose decreased to varying degrees during the aging process under different conditions, with the degree of change increasing as the aging time progressed. Notably, the relative intensity of hemicellulose characteristic peaks declined faster and more significantly than those of cellulose. For example, under 90% RH conditions, the peak intensity ratios of I_1730_/I_1505_ (Figure 6a) and I_1460_/I_1505_ (Figure 6b) showed significant changes by day 50, while the ratios of I_1370_/I_1505_ (Figure 6c) and I_1060_/I_1505_ (Figure 6d) exhibited noticeable changes only by day 75 or even day 100. By day 100, the peak intensity ratios of I_1730_/I_1505_, I_1460_/I_1505_, I_1370_/I_1505_, and I_1060_/I_1505_ under 90% RH conditions had decreased by 50.13%, 37.02%, 21.87%, and 35.24%, respectively, compared to their pre-aging state. This observation is consistent with previous research findings [47]. Samples aged in dry environments exhibited a similar phenomenon. For instance, after 100 days of aging under 10% RH, the peak intensity ratios of I_1730_/I_1505_, I_1460_/I_1505_, I_1370_/I_1505_, and I_1060_/I_1505_ decreased by 10.37%, 15.32%, 5.30%, and 11.73%, respectively, compared to their pre-aging state. These results indicate that hemicellulose is less stable than cellulose, degrading more readily and rapidly. This phenomenon was observed in all samples.

This phenomenon further explains the previously observed shift of the characteristic peak at 1060 cm^−1^ in the infrared spectrum (Figure 5e). In the early stages of aging (e.g., before 75 days), the primary degradation occurred in hemicellulose (Figure 6a,b). During this period, the characteristic peak at 1060 cm^−1^ shifted to higher wavenumbers due to the loss of hydroxyl groups and the weakening of hydrogen bonding [39,40]. Starting at around 100 days of aging, cellulose in the samples also began to degrade significantly (Figure 6c,d), resulting in the characteristic peak at 1060 cm^−1^ shifting to lower wavenumbers [39,40].

Furthermore, the rate of color change observed in the heatmaps (Figure 6) clearly demonstrates that samples aged in humid environments (70% RH and 90% RH) experienced more rapid and severe degradation of their primary chemical components due to mold infestation.

After 200 days of aging under different conditions, the relative peak intensities (I_1730_/I_1505_, I_1460_/I_1505_, I_1370_/I_1505_, and I_1060_/I_1505_) of the samples aged at 70% RH decreased by 54.77%, 52.92%, 45.88%, and 27.97%, respectively, compared to their pre-aging values. For samples aged at 90% RH, the reductions were even more significant, with decreases of 68.91%, 59.65%, 55.27%, and 53.52%, respectively. These findings indicate that in high-humidity environments, particularly when combined with mold invasion, the relative content of cellulose and hemicellulose declines significantly, with higher humidity levels resulting in greater degradation.

Samples aged at 10% RH and 30% RH for 200 days also showed reductions in peak intensities compared to their pre-aging values, with samples aged at 10% RH experiencing greater degradation. Although the degree of degradation was less severe than in samples aged under high humidity, the results suggest that prolonged exposure to dry environments can lead to desiccation and shrinkage, causing surface cracks (b,c and d,f). These irreversible phenomena contribute to the gradual degradation of cellulose and hemicellulose.

In contrast, samples aged at 50% RH for 200 days exhibited the smallest reductions in relative peak intensities, with decreases of only 11.16%, 15.93%, 3.31%, and 4.85% for I_1730_/I_1505_, I_1460_/I_1505_, I_1370_/I_1505_, and I_1060_/I_1505_, respectively. This minimal degradation highlights 50% RH as a relatively optimal humidity condition for the preservation of Palm Leaf Manuscripts.

### 3.4. XRD of the Samples During the Aging Process

**Figure 8:**
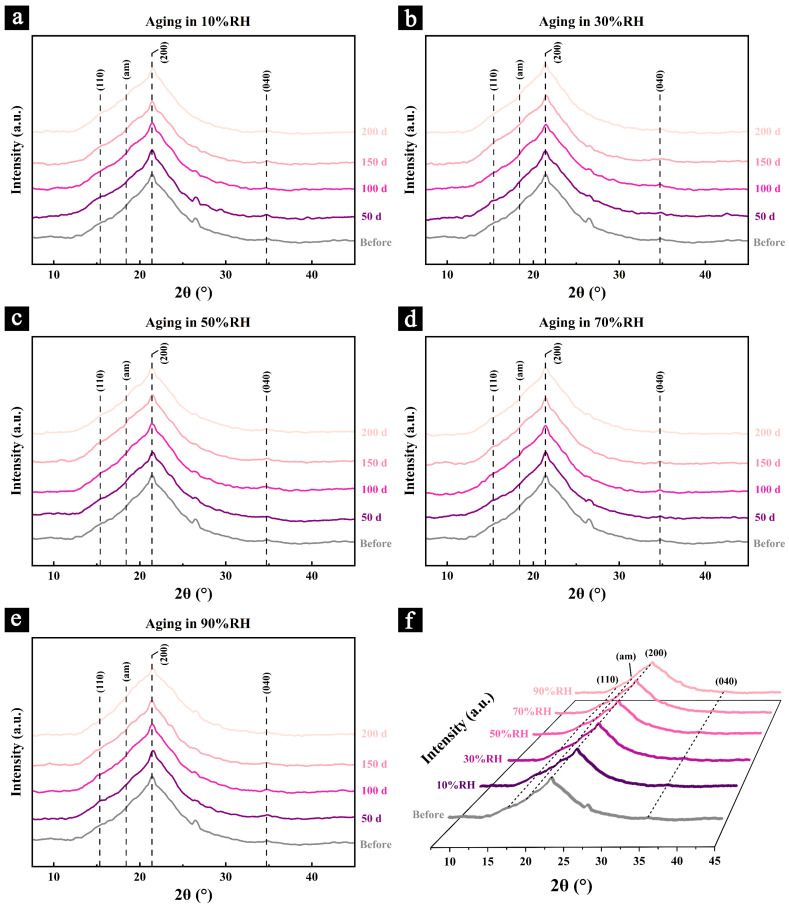
Changes in the XRD patterns of the samples during aging under different humidity conditions: 10% RH (**a**), 30% RH (**b**), 50% RH (**c**), 70% RH (**d**), and 90% RH (**e**); and the XRD patterns of the samples before and after 200 days of aging (**f**).

The X-ray diffraction (XRD) patterns of the samples during aging under different conditions and after 200 days of aging are presented in Figure 8.

The results reveal that all samples exhibit a prominent (200) diffraction peak near 22°, along with characteristic peaks corresponding to the 110 plane (approximately 15°) and the 040 plane (near 34°). These findings confirm that the cellulose crystalline structure in all samples remains of the cellulose I type, showing no significant changes due to aging under the various conditions. Additionally, some non-cellulose diffraction peaks, such as those observed at 26° and 29°, are likely attributed to other crystalline substances, including calcium (Ca) and silicon (Si), which were introduced during the preparation of the Palm Leaf Manuscripts [4].

Samples aged in relatively dry environments (10% RH, 30% RH, and 50% RH) (Figure 8a–c) showed a consistent trend of increased signal intensity and broadening of the amorphous region (am) over the aging period. This observation suggests the potential degradation of crystalline regions or the transition of cellulose from crystalline to amorphous states, contributing to a reduction in cellulose crystallinity.

In contrast, samples aged in humid environments (70% RH and 90% RH) exhibited a different trend in the amorphous region (am). During the early stages of aging (before 100 days), the signal intensity weakened, and the region narrowed. However, in the later stages of aging (after 100 days), the signal intensity increased, and the region broadened. These findings suggest that in humid environments, the initial degradation primarily targets non-crystalline components, such as hemicellulose and the amorphous regions of cellulose. As aging progresses, the crystalline regions of the cellulose also undergo significant degradation. This process may lead to a transient increase in the relative crystallinity of cellulose during the early stages of aging, followed by a notable decrease as the degradation of crystalline cellulose becomes more pronounced. These results are consistent with the findings from the infrared spectroscopy analysis (Figure 6).

Additionally, the characteristic peak of the 110 plane in samples aged in humid environments (70% RH and 90% RH) shifts to lower angles over time. Previous studies have suggested that excessive moisture absorption by cellulose can cause an expansion of the cellulose crystalline structure, leading to changes in lattice parameters and, consequently, a shift in the position of the 110 plane peak [48,49]. Moreover, cellulose degradation and the reduction in crystallinity may contribute to stress relaxation within the cellulose crystals, further causing the peak to shift to lower angles [50].

These observations indicate that aging, particularly under humid conditions, induces changes in the crystallinity of cellulose within the samples. This conclusion aligns with the degradation trends observed in other chemical and structural analyses.

**Figure 9:**
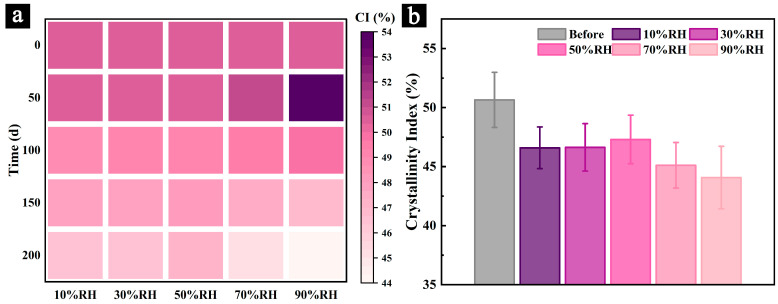
Relative crystallinity of cellulose in samples during aging under different humidity conditions (**a**) and after 200 days of aging (**b**).

To further investigate the changes in the chemical structure and primary chemical components of the samples, the relative crystallinity of the samples was calculated during aging under different conditions (Figure 9a) and after 200 days of aging (Figure 9b).

The results indicate that the relative crystallinity of cellulose in samples aged under relatively dry conditions (10% RH, 30% RH, and 50% RH) gradually decreases over time (Figure 9a). This reduction is likely attributed to the natural degradation of cellulose and its transition from crystalline regions to amorphous regions. Among these conditions, the decrease in relative crystallinity for samples aged at 50% RH is notably slower.

In contrast, the relative crystallinity of cellulose in samples aged under humid conditions (70% RH and 90% RH) initially increases and then decreases, with the reduction rate accelerating significantly after 100 days. The extent of this decline is considerably greater than that observed in samples aged under relatively dry conditions. These findings indicate that during the early stages of aging, high humidity and fungal infestation result in significant degradation of both cellulose and hemicellulose. However, non-crystalline components, such as hemicellulose and the amorphous regions of cellulose, degrade more rapidly and extensively (as evidenced by changes in the relative peak intensity ratios of infrared spectral features over time, as shown in Figure 6). Consequently, the relative crystallinity of cellulose increases significantly during the early phase of aging.

As aging progresses, particularly after 100 days, most of the hemicellulose is degraded, leaving cellulose and its crystalline regions as the primary targets for fungal degradation. At this stage, the relative crystallinity of cellulose begins to decrease markedly, aligning with the findings from the infrared spectral analysis (Figure 6).

After 200 days of aging under different conditions (Figure 9b), the relative crystallinity of cellulose decreased by 8.01%, 7.92%, 6.61%, 10.92%, and 13.11% for samples aged at 10% RH, 30% RH, 50% RH, 70% RH, and 90% RH, respectively. Among these, samples aged at 50% RH exhibited the smallest reduction in relative crystallinity, indicating greater structural stability under these conditions.

### 3.5. TGA Analysis of the Samples

**Figure 10:**
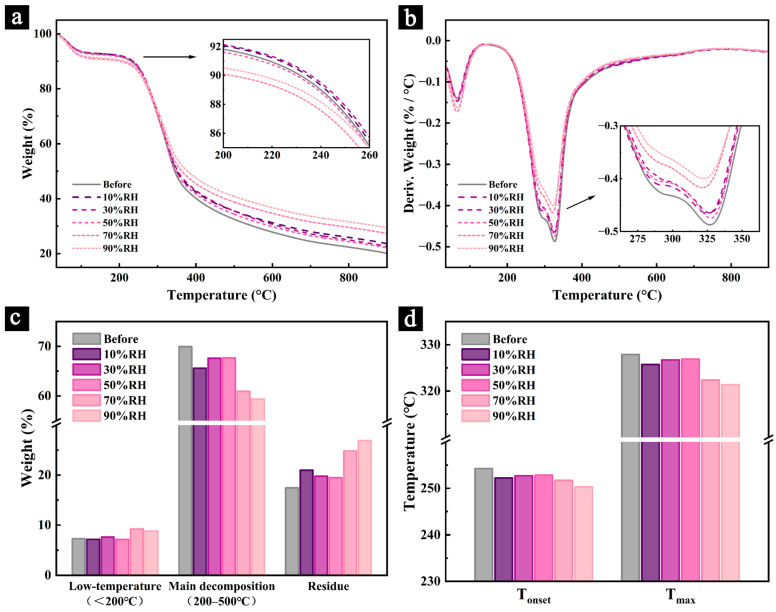
TG curves of samples before and after aging under different humidity conditions (**a**), DTG curves (**b**), mass ratios at each stage (**c**), and T_onset_ and T_max_ (**d**).

The TG and DTG curves, along with the related parameters of the samples after 200 days of aging under different conditions, are presented in Figure 10. The results reveal that, in both the low-temperature decomposition stage (<200 °C) and the primary pyrolysis stage (200–500 °C), samples aged in humid environments (70% RH and 90% RH) decomposed more rapidly. Additionally, these samples exhibited a significant increase in mass loss during the low-temperature decomposition stage compared to the pre-aging samples, indicating a higher content of water and volatile small molecules in samples aged under humid conditions.

For all samples aged under different conditions, the mass loss ratio during the primary pyrolysis stage decreased to varying extents compared to the pre-aging samples, while the residual char ratio increased. Among the primary chemical components, cellulose and hemicellulose typically decompose completely within the 200–500 °C range, leaving minimal residual char. In contrast, lignin, with its thermally stable aromatic ring structure, decomposes over a broader temperature range and exhibits significant carbonization characteristics at high temperatures, resulting in a higher residual char ratio [51,52]. These findings suggest that the cellulose and hemicellulose in all samples underwent varying degrees of degradation during aging (as evidenced by the reduced mass loss during the primary pyrolysis stage), leading to an increased relative content of lignin (indicated by the higher residual char ratio). Furthermore, samples aged in humid environments experienced more severe degradation due to mold activity, consistent with the results from the infrared spectroscopy analysis.

Additionally, the initial pyrolysis temperature (T_onset_) and the maximum pyrolysis temperature (T_max_) of the aged samples were both lower than those of the pre-aging samples, indicating a decline in the overall thermal stability of the samples after aging. This reduction in thermal stability was more pronounced in samples aged in humid environments. In contrast, samples aged at 50% RH exhibited relatively better thermal stability, emphasizing that 50% RH is a more favorable condition for preserving the thermal properties of Palm Leaf Manuscripts.

## 4. Conclusions

The results indicate that dry environments cause surface cracking on Palm Leaf Manuscripts, whereas humid environments lead to mold infestation, resulting in significant degradation of their primary chemical components. Hemicellulose was found to degrade more rapidly and extensively than cellulose. For instance, under 10% RH and 90% RH conditions, cellulose degradation reached 19.82% and 54.40%, respectively, while hemicellulose degradation reached 34.78% and 64.28%. These conditions also reduced the relative crystallinity of cellulose by 8.01% and 13.11%, respectively. Furthermore, the degradation of the primary chemical components contributed to a decline in the thermal stability of the manuscripts.

In contrast, samples aged at 50% RH exhibited no significant deterioration. Cellulose and hemicellulose degradation were limited to 4.08% and 13.55%, respectively, with the relative crystallinity of cellulose decreasing by only 6.61%. These samples also retained relatively good thermal stability, suggesting that 50% RH is an optimal humidity condition for the preservation of Palm Leaf Manuscripts.

This study provides a comprehensive understanding of the characteristics of Palm Leaf Manuscripts, contributing to the development of more effective preventive conservation strategies. However, it is important to note that the preservation of Palm Leaf Manuscripts often involves the interaction of multiple environmental factors, such as temperature and humidity. The identification of 50% RH as a relatively suitable condition is based on the findings of this study and represents a preliminary conclusion. Future research should investigate the combined effects of more complex environmental factors on the primary chemical components and other properties of Palm Leaf Manuscripts.

## Data Availability

The original contributions presented in this study are included in the article. Further inquiries can be directed to the corresponding author.
